# The Impact of Berberine on Intestinal Morphology, Microbes, and Immune Function of Broilers in Response to Necrotic Enteritis Challenge

**DOI:** 10.1155/2021/1877075

**Published:** 2021-10-19

**Authors:** Lin Yuan, Mengjie Li, Yingying Qiao, Haoyu Wang, Litong Cui, Mingfa Wang

**Affiliations:** ^1^Henan Key Laboratory of Farm Animal Breeding and Nutritional Regulation, Institute of Animal Husbandry and Veterinary Medicine, Henan Academy of Agricultural Sciences, Zhengzhou 450003, China; ^2^Bureau of Agriculture and Rural Affairs of Longting District, Kaifeng 475000, China; ^3^Sumy National Agrarian University, Faculty of Biology and Technology, Kiev 03115, Ukraine

## Abstract

The objective of this study was to explore the therapeutic effects of berberine on necrotic enteritis (NE) in broilers caused by *Clostridium perfringens*. A total of 240 1-day-old Arbor Acres chicks were divided into four groups, as negative controls (NC), positive controls (PC), berberine- (BER-) treated, or lincomycin- (LMY-) treated groups. Broilers were challenged with *C. perfringens* at 15-21 days of age, followed by BER or LMY supplied in drinking water for 7 days. Experimental results showed that *C. perfringens* infection significantly decreased growth performance and increased intestinal necrosis index and the number of *C. perfringens* present to 6.45 Log_10_CFU/g (*P* < 0.001). Proinflammatory cytokines in the ileum were significantly increased, but the expression of ileal tight junction proteins occludin and claudin-1 was significantly reduced. Both BER and LMY ameliorated some of these observations. Compared with the PC group, the number of *C. perfringens* in the cecum was significantly decreased following treatment (*P* < 0.001), and growth performance and small intestine morphology were similar to those of the NC group (*P* > 0.05). IL-1*β*, IL-6, and TNF-*α* levels as well as occludin and claudin-1 expression were also significantly improved (*P* < 0.05). BER has the potential to replace antibiotics for NE caused by *C. perfringens*.

## 1. Introduction

Necrotic enteritis (NE) is a serious enterotoxic disease of broiler chickens caused by *Clostridium perfringens*, costing up to 2 billion USD annually worldwide [1, 2]. Broilers are susceptible to NE at 2–6 weeks of age and manifest both clinical (acute) and subclinical (chronic) symptoms. Acute disease causes high mortality at 3–4 weeks of age, and chronic conditions can lead to reduced body weight gain and feed conversion efficiency. Reduced productivity due to subclinical symptoms is often difficult to detect, but up to 40% of commercial broilers may be affected by subclinical NE [3].

The etiology of NE includes *Clostridium perfringens*, nutrition, stress, and coccidiosis, of which *Clostridium perfringens* is the main factor [4]. Pathogenic strains of *C. perfringens* produce a variety of toxins, including more bacteriocin than nonpathogenic strains [5, 6]. This can inhibit other *C. perfringens* strains, and in conjunction with other virulence factors, contribute to intestinal necrosis. The early morphological changes of NE may be caused by lecithinase. Pathological damage first occurs in the basal and transverse domains of intestinal epithelial cells and then extends to the whole lamina propria. Both collagenase secreted by the host and lecithinase secreted by *C. perfringens* cause tissue damage [7]. NetB toxin is a necessary virulence factor produced by *Clostridium perfringens* to induce NE, with *in vitro* experiments showing that it causes perforation of chicken epithelial cells. Although its activity *in vivo* is unknown, it may cause necrotic damage via this mechanism in the intestine [8, 9]. As such, *C. perfringens* strains with host-specific virulence factors are essential for NE.

Traditional preventive and therapeutic measures include lincomycin (LMY) and virginiamycin added to feed. However, the European Union has banned various antibiotics as animal feed additives or growth promoters [1]. With chicken NE remaining a problem in poultry breeding, alternatives to antibiotics are urgently required. Berberine (BER) is a natural plant-derived drug with analgesic effects, antibacterial, and antidiarrhea effects in inflammatory bowel disease (IBD), effects against intestinal barrier dysfunction and spasmolysis, and regulates specific T-cell imbalance [10]. Dextran sulfate sodium (DSS) model studies of mice with intestinal damage have indicated that BER can improve body weight gain, support myeloperoxidase activity, and reduce inflammation and the level of proinflammatory cytokines in colon tissue caused by DSS [11]. At present, BER applications as feed additive for livestock and poultry have not been thoroughly investigated.

In this study, *C. perfringens* was used to construct an NE model of broilers as per Dahiya et al. [12], and berberine and LMY were compared in treatment efficacy.

## 2. Materials and Methods

### 2.1. Animal Experiments

All animal experiments were carried out in compliance with the ARRIVE guidelines and were conducted according to the Animal Care and Use Guidelines of the Animal Care Advisory Committee and approved by the Institutional Animal Care and Use Committee of the Henan Academy of Agricultural Sciences (protocol no. 20201018).

Two hundred and forty 1-day-old female Arbor Acres broilers were purchased from Kaifeng Xingda (Henan) Industrial Co., Ltd. and divided into four treatment groups based on the principle of uniform primary weight. Each treatment had six replicates, with 10 chickens per replicate. Test groups included a negative control (NC) group, a positive control (PC) group challenged with *C. perfringens*, a BER- treated after challenged with *C. perfringens* group, and a LMY- treated after challenged with *C. perfringens* group. The experiment was carried out for 28 days, during which broilers were fed *ad libitum*, free-flowing water, and provided with 24 h artificial light. During the first 7 days, ambient temperature was 35°C, and then decreased gradually to 22°C by the fourth week. Diets were configured according to NRC (Table 1) needs and provided as powdered feed.

### 2.2. Challenge with *C. perfringens*

*C. perfringens* CVCC2027 (China Veterinary Drug Administration Strain Preservation Center, China) was inoculated on brain heart infusion broth medium (Oxford Co., Ltd, UK) and cultured under anaerobic conditions at 37°C for 18 h, then inoculated into thioglycollate broth medium (Beijing Luqiao Technology Co., Ltd, China), and cultured under anaerobic conditions at 37°C for 24 h. On day 15 of broiler growth, each individual in the three *C. perfringens* infection groups was orally inoculated with 1 mL bacterial solution (2.0 × 10^8^ CFU/mL) once a day for 7 days. In the NC group, broilers were given an equal volume of 0.75% saline. On day 22 of broiler growth, for the treated groups, BER or LMY (Sigma-Aldrich, USA) was dissolved in drinking water at respective concentrations of 100 mg/L and 75 mg/L and provided in 1 L drinking containers for 7 days. When this volume had been consumed, nonmedicated drinking water was provided for the remainder of each day. At the end of the trial when chickens were 28 days old, one individual from each replicate group was slaughtered and sampled.

### 2.3. Growth Performance

On days 1, 14, 21, and 28, broilers were weighed after a 12 h fast, and average daily feed intake (ADFI), average daily gain (ADG), and feed conversion rate (FCR) were calculated for each replicate group. When calculating FCR, the weight of dead individuals was included.

### 2.4. Sample Collection and RNA Extraction

At the end of the 28 day period, one chicken was randomly selected from each replicate group, anesthetized by intravenous injection of 25 mg/kg of thiopental sodium (Takara, Dalian, China), after a few minutes, the broilers fell, general weakness, reaction disappeared, and then euthanized by carotid artery bloodletting. After the whole experiment, the surviving broilers were euthanized, disinfected strictly, and buried deeply. We extracted total RNA from approximately 50 mg of ileal tissue using TRIzol kit (Takara, Dalian, China) according to the manufacturer's instructions. The RNA concentration was determined by absorbance at 260 nm using a Nanodrop 1000 (NanoDrop Technologies, Wilmington, US). We evaluated RNA purity by measuring the OD260/OD280 ratio. The RNA samples typically had an OD260/OD280 ratio between 1.9 and 2.0. Pathological changes in the small intestine were observed, and intestinal lesion scoring (ILS) was applied according to the severity of intestinal injury on a 0-4 point scale [12]. A 3 cm segment was washed with 0.75% saline to determine cytokines. Another segment was fixed with 4% paraformaldehyde to measure villus height (VH) and crypt depth (CD). Cecum chyme was collected and used to determine the number of bacteria.

### 2.5. Cecum Microflora

*C. perfringens*, *Escherichia coli*, and *Lactobacillus* spp. were cultured using tryptose sulfite cycloserine agar base, MacConkey media, and MRS media (Beijing Luqiao Technology Co., Ltd, China), respectively. *C. perfringens* and *Lactobacillus* spp. were cultured under anaerobic conditions at 37°C, and *E. coli* was cultured under aerobic environment at 37°C and counted after 24 h.

### 2.6. Intestinal Morphology

The duodenum, jejunum, and ileum were embedded in paraffin and serial sections were stained with hematoxylin-eosin (Nanjing Jiancheng Biological Engineering Research Institute Co. Ltd, China). The morphology of each segment was observed under the microscope of Olympus CK 40. VH and CD were measured based on ten intact villi at 400×, and VH:CD was calculated.

### 2.7. Ileal Cytokines

The ileum segment was homogenized with 0.1 M PBS and then centrifuged at 3000 g for 10 minutes. Supernatant protein concentration was measured using a BCA protein quantification kit (Thermo, USA). IL-1*β*, IL-6, and TNF-*α* contents were determined using a chicken-source ELISA kit (Cusabio Biotech Co. Ltd, Wuhan, China) following the manufacturers' instructions.

### 2.8. Relative Expression of Ileal Tight Junction Protein-Related Gene mRNA

A first-stand complementary DNA (cDNA) was reversed immediately with 1 *μ*g of total RNA using a Prime Script TM RT reagent kit (Takara, Japan) according to the manufacturer's instructions. All cDNA samples were stored at −80°C before further analysis. Occludin, claudin-1, claudin-2, ZO-1, and *β*-actin cDNA primers were designed based on the GenBank sequences D21837.1, AY750897, NM_001277622, XM_413773, and NM_205518, respectively (Table 2), and relative mRNA expression was measured by comparison threshold method. Analysis of these targets was performed using qPCR with a SYBR Premix Ex Tap kit (Takara, Japan) and a 7500 Real-Time PCR System (Applied Biosystems) according to a program of heating at 95°C for 30 s, followed by 40 cycles of denaturation at 95°C for 5 s and annealing at 60°C for 34 s. After amplification, the specificity of the product was verified using analysis of the dissolution curve and agarose gel electrophoresis. The quantity of ileal tight junction protein mRNA in each sample was normalized to *β*-actin. The cDNA of the digestive enzymes was quantified using relative standard-curve methods. Because the amplification efficiencies of the target and references genes were slightly different, the quantification of the gene copy number was obtained from different standard curves for the target and reference genes. The average value obtained for the NC sample was defined as 1, and the experimental results are expressed as a percentage of those obtained for the control group.

### 2.9. Statistical Analyses

Differences between groups were compared using ANOVA in SPSS Version 20.0 for Windows (SPSS Inc., Chicago, Illinois, USA). Significant differences between the treatments, defined as *P* < 0.05, were analyzed using Student-Newman-Keuls multiple comparison. Results are expressed as means and standard deviation for each group.

## 3. Results

### 3.1. Performance

The production performance results of broilers in each test group are shown in Table 3. Prior to *C. perfringens* challenge (1-14 days), growth performance was similar between test groups (*P* > 0.05). *C. perfringens* significantly reduced the ADG (by 10.16%-11.51%) and FCR (by 6.72%-8.21%) of broilers during the challenge (*P* < 0.05, 15-21 days). Two broilers died of NE during this phase, but no other mortality occurred. During the treatment period (22-28 days), ADG and FCR of broilers in the PC group were still poorer (by 13.10% and 12.03%, *P* < 0.05) than in the NC group. There was no significant difference in growth performance between the BER and LMY treatment groups (*P* > 0.05).

### 3.2. Intestinal Lesion Score and Morphology

No necrotic damage to the small intestine was observed in the NC group (Table 4). *C. perfringens* challenge significantly increased ILS scores to 2.25 (*P* < 0.001). After treatment with BER or LMY, ILS decreased significantly to 0.5 and 0.33 (*P* < 0.001).

Compared with the NC group, VH and VH/CD of the duodenum, jejunum, and ileum in the PC group decreased in varying degrees (*P* > 0.05). Compared with the PC group, duodenal VH in the BER and LMY treatment groups was significantly increased by 25.00% and 28.96% (*P* < 0.001), and VH/CD was significantly increased by 25.99% (*P* = 0.004) and 32.82% (*P* = 0.001). LMY treatment significantly improved the VH/CD of jejunum by 17.72% (*P* = 0.014) and significantly reduced the ileum CD by 11.35% (*P* = 0.027).

### 3.3. Cecum Microflora

**Figure 1:**
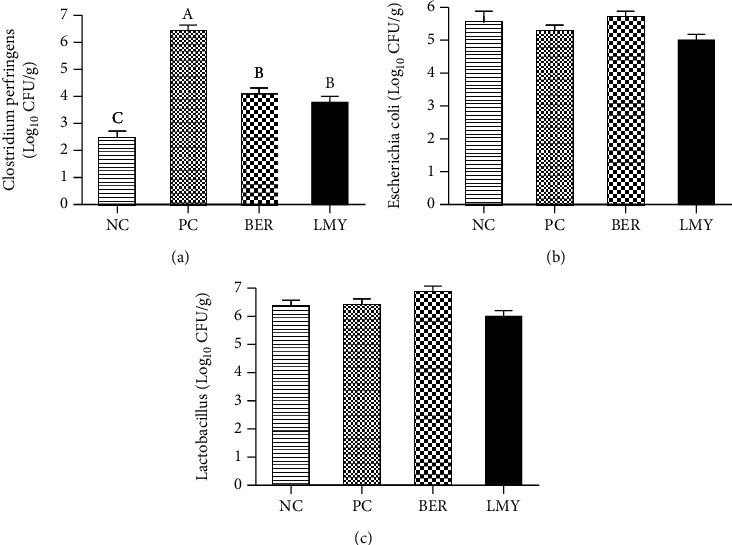
Effect of BER and LMY on cecum microflora of broilers challenged with *C. perfringens*. (a) *Clostridium perfringens*. (b) *Escherichia coli*. (c) *Lactobacillus*. Values with no superscript letter or the same superscript letter are not significantly different (*P* > 0.05); those with different superscript letters are significantly different (*P* < 0.05). NC: negative control; PC: positive control; BER: berberine-treated; LMY: lincomycin-treated.

*C. perfringens* challenge significantly increased the number of *C. perfringens* to 6.45 Log_10_CFU/g in the cecum (*P* < 0.001; Figure 1). Compared with the PC group, BER and LMY significantly decreased the number of *C. perfringens* to 4.14 and 3.81 Log_10_CFU/g (*P* < 0.001). Challenge did not affect the number of *E. coli* and *Lactobacillus* spp. (*P* > 0.05). Compared with the LMY treatment group, the number of *Lactobacillus* in the BER treatment group was higher (*P* = 0.054), which may indicate greater efficacy of BER compared with LMY in treating NE.

### 3.4. Ileal Cytokines

**Figure 2:**
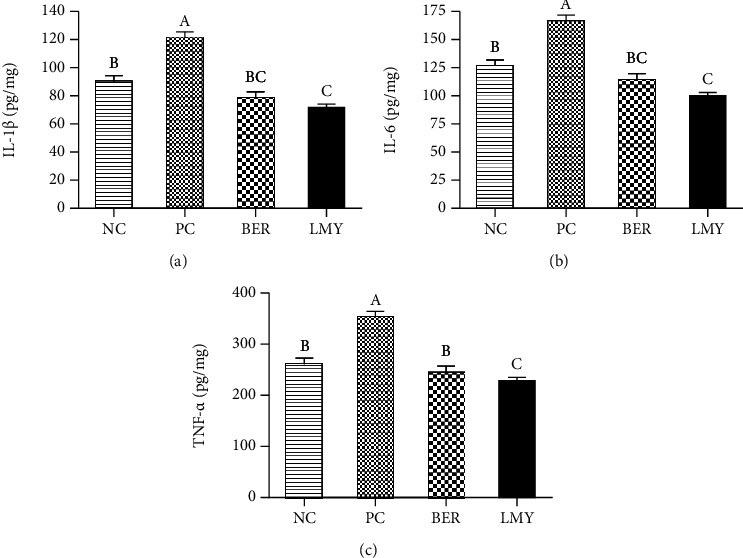
Effect of BER and LMY on ileal cytokines of broilers challenged with *C. perfringens*. (a) IL-1*β*. (b) IL-6. (c) TNF-*α*. Values with no superscript letter or the same superscript letter are not significantly different (*P* > 0.05); those with different superscript letters are significantly different (*P* < 0.05). NC: negative control; PC: positive control; BER: berberine-treated; LMY: lincomycin-treated.

*C. perfringens* challenge significantly raised levels of IL-1*β*, IL-6, and TNF-*α* by 34.15%, 33.10%, and 33.82% in the ileum (*P* < 0.001; Figure 2). These were significantly decreased under both BER and LMY treatment (*P* < 0.001). Compared with the NC group, LMY treatment significantly reduced ileal IL-*β* and IL-6 by 20.22% (*P* = 0.003) and 20.61% (*P* = 0.002), while BER also reduced these measured, but to a lesser degree. There was no significant difference in ileal cytokines levels among two treatment groups (*P* > 0.05).

### 3.5. Relative Expression of Ileal Tight Junction Protein-Related Gene mRNA

**Figure 3:**
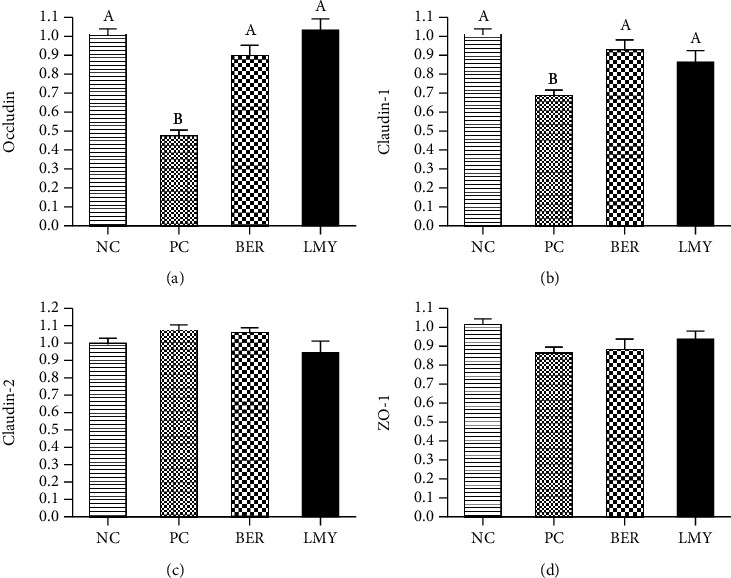
Effect of BER and LMY on ileal tight junction protein-related gene expression in broilers challenged with *C. perfringens*. (a) *Occludin*. (b) *Claudin-1*. (c) *Claudin-2*. (d) *ZO-1*. Values with no superscript letter or the same superscript letter are not significantly different (*P* > 0.05); those with different superscript letters are significantly different (*P* < 0.05). NC: negative control; PC: positive control; BER: berberine-treated; LMY: lincomycin-treated.

Occludin and claudin-1 were significantly less expressed by 53% and 32% in the PC group compared to the NC group (*P* < 0.001, Figure 3). Compared with the PC group, both BER and LMY treatment significantly improved relative expression of occludin to 0.88 and 1.02 (*P* < 0.001), and claudin-1 improved to 0.92 (*P* = 0.003) and 0.86 (*P* = 0.022). There was no significant difference in the expression of claudin-2 and ZO-1 between the groups (*P* > 0.05).

## 4. Discussion

*C. perfringens* challenge caused significant pathological manifestations of necrotic enteritis, including intestinal hemorrhagic lesions, deterioration of feed utilization efficiency, and a significant increase in the number of *C. perfringens* in the cecum, consistent with previous reports [13, 14]. Intestinal injury is an important indicator of the severity of NE, and in experimental NE, the number of *C. perfringens* is positively correlated with the severity of necrotic injury [3]. Initial pathological changes are caused by several collagenases secreted by *C. perfringens* that can degrade collagen in loose connective tissue [7, 15]. In addition, the toxin secreted by *C. perfringens* is also an important factor causing NE [16]. Inflammatory injury destroys the normal functional form between intestinal epithelial cells and lamina propria, causing severe inflammatory reactions and worsening feed utilization, thereby reducing broiler growth performance [17]. In this study, *C. perfringens* challenge significantly reduced growth performance of broilers and caused intestinal damage. After BER treatment, ADG and FCR were not significantly different from those of the NC group, and intestinal damage was significantly improved on par with the therapeutic effect of LMY. This indicates that BER could be an effective support for broiler growth performance by guarding against intestinal damage caused by *C. perfringens*.

BER has been shown to have antibacterial, anti-inflammatory, antihyperlipidemic, antihyperglycemic, antioxidant, and immunoregulatory effects, as well as being effective in the treatment of some tumors [18]. It can directly interact with bacterial lipopolysaccharide (LPS), peptidoglycan, and surface polysaccharides and proteins, thereby contributing to a broad-spectrum antibacterial effect against a variety of drug-resistant bacteria [19]. After oral administration, BER remains in the intestine because it is not easily absorbed, resulting in a high local concentration, and creating a bacteriostatic effect. When enteritis occurs, the number of *E. coli* and *Enterococcus* spp. increases while the number of *Lactobacillus* spp. and *Bifidobacterium* spp. decreases; BER can improve these changes [10]. Berberine has an inhibitory effect on resident bacteria in the human intestinal tract, including a stronger effect on harmful bacteria such as *C. putrefaciens* and a weaker effect on beneficial bacteria such as *Lactobacillus* spp. [20]. Administration of BER in C57BL/6 mice with colitis induced by *C. difficile* infection effectively inhibited the reproduction of *Enterobacteriaceae*, counteracted the side effects of vancomycin, adjusted the intestinal microflora, prevented colitis recurrence, and improved survival rate [21]. The antibacterial effect of BER is based on its influence on bacterial metabolism and enhancement of calcium ion permeability of the intestinal mucosal epithelial cell wall. When enteritis occurs, water and electrolytes leak from the gaps between the mucosal cells leading to diarrhea. Thus, BER can directly affect the exosmosis of water and electrolytes induced by bacterial toxins in the intestine.

Intestinal morphology is an indicator of intestinal health status. High VH indicates increased intestinal mucosal absorption area, a greater population of mature intestinal epithelial cells, and greater absorption capacity, while lower VH indicates fewer mature intestinal epithelial cells and lower digestive and absorption capacity [22]. Increasing CD indicates that the body may reconstruct villi by accelerating the rate of cell renewal to resist damage caused by pathogenic bacteria or toxins [23]. In this study, *C. perfringens* infection not only caused visible damage but also significantly reduced the VH and VH/CD of all segments of small intestine, consistent with previous reports [24]. Intestinal VH/CD is regarded as an important measure of intestinal health and epithelial cell repair [25]. In this study, addition of both BER and LMY significantly increased VH and VH/CD in the duodenum, thereby indicating that both agents can support the repair of intestinal injury and enhance intestinal absorption.

Under intestinal bacterial infection, the host immune response causes inflammatory cells to migrate to the site. If this response cannot be controlled, it will cause tissue damage. The occurrence of NE is closely related to the cytokine response, with this response dominated by the T-cell differentiation pattern of the initiating disease. Proinflammatory factors such as IL-1*β*, IL-6, and TNF-*α* can mediate the occurrence of NE [26]. Cytokines produced by inflammatory cells are toxic to surrounding cells, causing damage and cell decomposition [27, 28]. Proliferation of *C. bacterium* causes a proinflammatory immune response [29]. The injection of *C. perfringens β*-toxin into mice can increase the expression of TNF-*α* and IL-1*β* [30]. In our study, BER and LMY significantly reduced ileal proinflammatory cytokines IL-1*β*, IL-6, and TNF-*α*. BER can alleviate the symptoms of NE by regulating the balance of immune response and reducing the levels of IFN-*γ*, IL-17, IL-6, IL-1*β*, and TNF-*α* in colonic mucosal epithelial cells and serum [31]. In Caco-2 cells, BER can reverse barrier dysfunction induced by TNF-*α* and IL-1*β* and reduce cell permeability and repair tight junction damage between cells [32], consistent with research results in HT-29/B6 cells. BER can effectively inhibit endoplasmic reticulum stress induced by TNF-*α*, IFN-*γ*, and tunicamycin in Caco-2 cells [33]. In rats, BER inhibits increasing TNF-*α* and IL-6 levels induced by multimicrobial sepsis in the intestinal mucosal barrier and increases the level of tight junction proteins and permeability of epithelial cells [34]. Therefore, BER can inhibit the secretion of proinflammatory cytokines, help to restore the intestinal mucosal epithelial barrier function, and inhibit the inflammatory response, thereby mediating the recovery of the intestinal mucosa.

Intestinal mucosal epithelial cells and the tight junctions between them form a mechanical barrier to prevent the invasion of antigens. The main tight junction proteins include occludin, claudins, and ZO-1 [35]. Tight junction disorders lead to increased intestinal permeability and decreased intestinal epithelial barrier function. *C. perfringens* infection significantly downregulated the mRNA expression of occludin and claudin-1 in the small intestine, as reported in previous studies [25]. In our experiment, both LMY and BER treatment significantly upregulated expression of occludin and claudin-1 in response to *C. perfringens* challenge. Another study showed that BER could repair the reorganize the tight junction proteins in the epithelial and membrane microregions of the colon in mice inflicted with endotoxemia by intraperitoneal injection of LPS [36]. Intraperitoneal injection of BER can also improve the intestinal mucosal epithelial barrier injury induced by peritoneal air exposure [37]. For mice with DDS-induced intestinal damage and colitis, BER can reduce colon tissue damage, upregulate the expression of ZO-1 and occludin, upregulate the expression of antiapoptotic proteins, and downregulate the expression of apoptotic proteins [38]. In the present study, there was no significant difference in the expression of claudin-2 and ZO-1 between the groups, while the PC group had the highest expression of claudin-2. Claudin-2 is a channel protein, and its increased content indicates increased intestinal permeability. Previous studies have shown that the expression of claudin-2 increases in enteritis, while inhibition of claudin-2 permeability has therapeutic effects [39, 40].

## 5. Conclusion

Our research indicates that NE induced by *C. perfringens* in broiler chickens significantly increased ILS and ileal proinflammatory cytokines and decreased duodenal VH and VH/CD. It also decreased the expression of intestinal tight junction protein-related genes occludin and claudin-1 and significantly reduced growth performance. Treatment with LMY and BER significantly reduced the number of *C. perfringens* in the cecum, growth performance was not significantly different from that of the NC group, improved intestinal morphology, inflammatory cytokines, and cell tight junction protein expression, such that these measures did not differ significantly from the NC group. BER showed the same effect against to *C. perfringens* infection as LMY.

## Figures and Tables

**Table 1:** Diet compositions and nutrient levels.

Ingredients (%)	
Corn	59.79
Soybean meal	34.28
Vegetable oil	2.20
Dicalcium phosphate	1.50
Limestone	1.40
Table salt	0.30
*DL*-methionine	0.20
Mineral premix^1^	0.10
*L*-lysine	0.10
Choline chloride	0.10
Vitamin premix^2^	0.03
Nutrient levels	
ME (kcal/kg)	2988.64
Crude protein	21.62
Met (%)	0.54
Lys (%)	1.16
Calcium (%)	1.02
Nonphytate phosphorus (%)	0.46

^1^Provided per kg of diet: vitamin A, 12 500 IU; vitamin D_3_, 3 000 IU; vitamin E, 20 IU; vitamin K_3_, 2.5 mg; vitamin B_1_, 2 mg; vitamin B_2_, 6 mg; vitamin B_6_, 3.5 mg; vitamin B_12_, 0.02 mg; pantothenic acid, 12 mg; niacin, 35 mg; folic acid, 1.5 mg; biotin, 0.2 mg. ^2^Provided per kg of diet: Cu (copper sulfate), 10 mg; Fe (ferrous sulfate), 60 mg; Zn (zinc sulfate), 75 mg; Mn (manganese sulfate), 100 mg; I (potassium iodide), 0.7 mg; Se (sodium selenite), 0.15 mg.

**Table 2:** Primer pairs for tight junction genes from broilers.

Primer	Primer sequence (5′-3′)	Accession no.
Occludin	F: ACGGCAGCACCTACCTCAA	D21837.1
	R: GGGCGAAGAAGCAGATGAG	
Claudin-1	F: CATACTCCTGGGTCTGGTTGGT	AY750897
	R: GACAGCCATCCGCATCTTCT	
Claudin-2	F: CAAGGACCGAGTGGCAGTG	NM_001277622
	R: TTTGATGGAGGGCTGAGGA	
ZO-1	F: CTTCAGGTGTTTCTCTTCCTCCTC	XM_413773
	R: CTGTGGTTTCATGGCTGGATC	
*β*-Actin	F: GAGAAATTGTGCGTGACATCA	NM_205518
	R: CCTGAACCTCTCATTGCCA	

**Table 3:** Effect of BER and LMY on performance of broilers challenged with *C. perfringens.*

Parameter	NC	PC	BER	LMY
1–14 days				
ADFI (g/d)	34.06 ± 1.25	35.33 ± 1.22	35.05 ± 1.09	34.08 ± 1.26
ADG (g/d)	28.74 ± 0.83	29.59 ± 0.82	29.34 ± 0.88	29.06 ± 1.21
FCR	1.19 ± 0.02	1.19 ± 0.01	1.20 ± 0.03	1.17 ± 0.03
15–21days				
ADFI (g/d)	81.30 ± 4.67	77.52 ± 3.37	77.41 ± 3.24	78.25 ± 3.31
ADG (g/d)	60.62 ± 3.85^a^	54.31 ± 2.32^b^	53.64 ± 4.15^b^	54.46 ± 4.18^b^
FCR	1.34 ± 0.02^b^	1.43 ± 0.03^a^	1.45 ± 0.06^a^	1.44 ± 0.06^a^
22–28 days				
ADFI (g/d)	109.74 ± 5.65	106.72 ± 3.43	112.13 ± 4.57	110.15 ± 4.29
ADG (g/d)	69.63 ± 3.31^a^	60.51 ± 2.84^b^	67.36 ± 3.22^ab^	66.81 ± 3.76^ab^
FCR	1.58 ± 0.07^b^	1.77 ± 0.13^a^	1.67 ± 0.10^ab^	1.65 ± 0.10^ab^

Each value represents the mean ± SD of six replicates. Values with no superscript letter or the same superscript letter are not significantly different (*P* > 0.05); those with different superscript letters are significantly different (*P* < 0.05). NC: negative control; PC: positive control; BER: berberine-treated; LMY: lincomycin-treated; ADG: average daily gain; ADFI: average daily feed intake; FCR: feed conversion ratio.

**Table 4:** Effect of BER and LMY on intestinal lesion score and morphology of broilers challenged with *C. perfringens.*

Parameter	NC	PC	BER	LMY
ILS	0.17 ± 0.26^b^	2.25 ± 0.99^a^	0.50 ± 0.45^b^	0.33 ± 0.52^b^
Duodenum				
VH (*μ*m)	912.88 ± 42.92^b^	881.46 ± 64.10^b^	1101.84 ± 64.78^a^	1136.73 ± 63.74^a^
CD (*μ*m)	188.87 ± 15.89	195.48 ± 12.16	193.96 ± 13.76	189.58 ± 12.78
VH/CD	4.87 ± 0.58^b^	4.54 ± 0.60^b^	5.72 ± 0.66^a^	6.03 ± 0.70^a^
Jejunum				
VH (*μ*m)	808.48 ± 69.60	778.99 ± 65.77	819.97 ± 56.45	848.02 ± 67.40
CD (*μ*m)	158.75 ± 9.82	171.81 ± 15.15	167.87 ± 11.55	158.48 ± 16.90
VH/CD	5.09 ± 0.63^ab^	4.57 ± 0.59^b^	4.89 ± 0.30^ab^	5.38 ± 0.53^a^
Ileum				
VH (*μ*m)	589.94 ± 62.60	576.35 ± 37.11	608.62 ± 67.74	571.72 ± 55.61
CD (*μ*m)	136.18 ± 11.88^ab^	147.70 ± 11.75^a^	136.42 ± 12.98^ab^	130.94 ± 11.88^b^
VH/CD	4.37 ± 0.68	3.92 ± 0.40	4.51 ± 0.76	4.40 ± 0.63

Each value represents the mean ± SD of six replicates. Values with no superscript letter or the same superscript letter are not significantly different (*P* > 0.05); those with different superscript letters are significantly different (*P* < 0.05). NC: negative control; PC: positive control; BER: berberine-treated; LMY: lincomycin-treated; ILS: intestinal lesion score; VH: villus height; CD: crypt depth; VH/CD: villus height: crypt depth.

## Data Availability

All relevant data are within the paper and its supporting information files.

## Supplementary Materials

Supplementary Materials The raw data of performance, intestinal morphology, cecum microflora, ileal cytokines, and mRNA relative ratio are provided in the supplemental file.
