# Supplementary material for: The Impact of Berberine on Intestinal Morphology, Microbes, and Immune Function of Broilers in Response to Necrotic Enteritis Challenge
Source: Biomed Res Int. 2021 Oct 19;2021:1877075. doi: 10.1155/2021/1877075 (PMC8548107; doi:10.1155/2021/1877075)
Supplement: Supplementary Materials — The raw data of performance, intestinal morphology, cecum microflora, ileal cytokines, and mRNA relative ratio are provided in the supplemental file. [file 1877075.f1.pdf]

Performance

	1-14 d			15-21 d			22-28 d		
	**ADG(g/d)**	**ADFI(g/d)**	**FCR**	**ADG(g/d)**	**ADFI(g/d)**	**FCR**	**ADG(g/d)**	**ADFI(g/d)**	**FCR**
1-1	28.65	35.17	1.227574171	56.47	77.35	1.369753852	66.99	101.92	1.521421108
1-2	27.38	32.29	1.179327977	59.93	79.11	1.320040047	72.52	115.69	1.59528406
1-3	28.53	32.86	1.151770067	55.95	75.27	1.345308311	73.17	107.42	1.468088014
1-4	28.78	34.01	1.181723419	64.26	86.36	1.343915344	72.04	114.33	1.587034981
1-5	29.17	34.74	1.190949606	64.99	85.68	1.31835667	67.56	113.81	1.684576673
1-6	29.92	35.3	1.179812834	62.14	84.04	1.352429997	65.51	105.26	1.606777591
2-1	29.76	36.04	1.211021505	52.39	75.38	1.438824203	63.45	104.67	1.64964539
2-2	29.34	34.31	1.16939332	54.73	76.21	1.392472136	59.37	111.79	1.882937511
2-3	28.32	33.53	1.183968927	57.06	81.84	1.434279706	57.88	105.01	1.814270905
2-4	30.86	36.88	1.19507453	52.79	74.07	1.403106649	63.34	103.62	1.63593306
2-5	29.77	35.76	1.201209271	57.01	81.69	1.432906508	56.92	110.34	1.93851019
2-6	29.51	35.48	1.202304304	51.88	75.95	1.463955281	62.11	104.88	1.68861697
3-1	29.36	36.01	1.226498638	48.9	74.35	1.520449898	65.34	112.33	1.71916131
3-2	29.51	34.3	1.162317858	54.23	76.42	1.409183109	63.75	111.56	1.749960784
3-3	30.74	36.24	1.178919974	55.15	78.03	1.41486854	65.52	114.74	1.751221001
3-4	29.6	35.32	1.193243243	57.92	81.62	1.409185083	71.13	119.31	1.677351329
3-5	28.33	35.09	1.238616308	48.27	73.59	1.52454941	71.48	106.75	1.493424734
3-6	28.47	33.33	1.170706006	57.38	80.47	1.402405019	66.96	108.06	1.613799283
4-1	28.81	33.52	1.163484901	58.49	81.56	1.394426398	65.24	107.14	1.642244022
4-2	29.25	35.48	1.212991453	50.62	75.11	1.483800869	71.79	105.37	1.467753169
4-3	29.82	33.97	1.139168343	49.96	74.19	1.48498799	63.67	109.69	1.722789383
4-4	27.34	32.25	1.179590344	51.44	77.58	1.508164852	62	108.78	1.754516129
4-5	30.83	35.56	1.153421992	58.53	82.35	1.406970784	69.12	117.39	1.698350694
4-6	28.33	33.71	1.189904695	57.71	78.69	1.363541847	69.03	112.54	1.630305664

Intestinal morphology

	ILS	Duodenum VH(μm)	CD(μm)	VH/CD	Jejunum VH(μm)	CD(μm)	VH/CD	Ileum VH(μm)	CD(μm)	VH/CD
1-1	0	899.84	202.99	4.4329277	712.63	155.94	4.5698987	596.19	129.81	4.5927895
1-2	0.5	918.03	199.26	4.6071966	837.54	173.08	4.839034	654.07	153.39	4.2640981
1-3	0.5	947.71	172.45	5.4955639	867.69	151.14	5.7409686	566.84	130.83	4.3326454
1-4	0	908.22	169.72	5.3512845	871.22	147.33	5.9133917	490.64	144.9	3.3860594
1-5	0	840.36	205.74	4.0845728	729.8	167.62	4.3538957	658.93	120.05	5.4887963
1-6	0	963.09	183.03	5.2619243	832	157.37	5.14	572.97	138.07	4.1498515
2-1	2	913.95	199.44	4.5825812	712.3	189.06	3.767587	543.25	139.7	3.8886901
2-2	3	826.46	204.35	4.0443357	800.79	155.3	5.156407	622.43	133.21	4.6725471
2-3	3	895.35	191.76	4.6691176	816.83	178.34	4.5801839	604.42	163.24	3.7026464
2-4	0.5	890.79	187.02	4.7630735	789.97	187.85	4.2053234	528.99	149.37	3.5414742
2-5	2	791.14	211.87	3.7340822	864.06	161.72	5.3429384	561.97	141.46	3.9726424
2-6	3	971.06	178.42	5.4425513	690	158.56	4.351665	597.04	159.22	3.7497802
3-1	1	1186	177.03	6.6994295	786.74	181.51	4.3344168	555.39	126.99	4.373494
3-2	0.5	1066.71	196.74	5.4219274	888.28	179.25	4.955537	578.91	145.56	3.9771228
3-3	0	1107.36	205.33	5.3930746	896.45	173.22	5.1752107	702.74	119.79	5.8664329
3-4	0	1034.69	184.51	5.6077719	782.37	157.65	4.9627022	540.05	148.06	3.6475078
3-5	1	1171.57	186.65	6.2768283	773.86	160.54	4.8203563	682.16	150.09	4.5450063
3-6	0.5	1044.7	213.47	4.8938961	792.11	155.02	5.1097278	592.44	128.02	4.6277144
4-1	0	1184.36	171.61	6.9014626	825.71	174.4	4.7345757	568.61	129.51	4.3904718
4-2	1	1156.72	202.44	5.7138905	825.64	164.09	5.031629	519.16	129.19	4.0185773
4-3	0	1229.31	176.83	6.9519312	774.45	146	5.3044521	519.72	149.89	3.4673427
4-4	0	1065.08	190.78	5.5827655	930.19	178.53	5.2102728	620.21	138.17	4.4887457
4-5	0	1089.5	201.5	5.4069479	799.35	134.95	5.9233049	655.55	122.93	5.3327097
4-6	1	1095.41	194.32	5.6371449	932.78	152.88	6.1013867	547.04	115.94	4.7183026

Cecum microflora(Log10CFU/g)

	C. perfringens	*E. coli*	*Lactobacillus* spp.
1-1	2.14	5.14	7.13
1-2	3.23	4.58	5.89
1-3	2.06	6.71	5.83
1-4	2.79	5.84	6.74
1-5	2.82	5.99	6.05
1-6	2.26	4.87	6.1
2-1	6.89	5	6.3
2-2	5.77	5.83	5.57
2-3	7.14	6.12	6.02
2-4	6.17	5.03	5.45
2-5	6.69	4.76	7.05
2-6	6.03	4.54	6.93
3-1	4.89	6.16	6.19
3-2	3.52	5.12	5.95
3-3	4.3	5.07	7.43
3-4	3.89	6.49	7.34
3-5	3.68	5.28	5.63
3-6	4.56	6.02	7.39
4-1	4.45	4.81	5.34
4-2	4.47	4.76	6.27
4-3	3.38	5.55	4.52
4-4	4.21	4.28	6.3
4-5	3.16	4.6	6.2
4-6	3.19	5.69	6.35

Ileal cytokines（pg/mg）

	IL-1β	IL-6	TNF-α
1-1	79.44	118.71	275
1-2	82.57	133.04	278.11
1-3	100.05	109.51	261.38
1-4	98.34	135	240.65
1-5	81.62	141.66	289.42
1-6	97.29	117.44	239.5
2-1	114.89	153.26	378.91
2-2	107.83	168.66	332.56
2-3	130.45	171.41	363.49
2-4	118	150.84	344.1
2-5	139.75	179	370.54
2-6	112.6	182.17	330.28
3-1	69.24	105.72	254.07
3-2	87.07	100.07	259.11
3-3	84.2	109.78	266.95
3-4	72.69	134.15	237.34
3-5	90.56	107.66	214.21
3-6	78.93	128.08	231
4-1	66.17	104.19	193.58
4-2	64.96	87.1	245.3
4-3	77.2	109.67	203.39
4-4	78.18	86.45	210.88
4-5	66.44	117.58	248.74
4-6	77.32	94.68	224.51

mRNA relative ratio

	Occludin	Claudin-1	Claudin-2	ZO-1
1-1	0.87	0.94	1.12	1.05
1-2	1.09	0.89	1.07	0.93
1-3	0.94	1.06	0.9	1.02
1-4	1	1	0.95	1.01
1-5	1.01	1.07	0.86	0.88
1-6	1.11	1.03	1.09	1.12
2-1	0.52	0.86	1.02	0.75
2-2	0.54	0.81	1.09	0.91
2-3	0.44	0.62	1.13	0.99
2-4	0.36	0.54	0.91	0.73
2-5	0.44	0.59	1.07	1.01
2-6	0.51	0.65	1.2	0.75
3-1	0.97	0.7	1.07	0.71
3-2	1.01	1.04	0.93	1.05
3-3	0.78	1.07	1.14	0.77
3-4	1.04	1.02	1.11	0.94
3-5	0.71	0.91	1.1	0.79
3-6	0.78	0.8	0.98	1.02
4-1	1.02	0.87	0.9	1.06
4-2	1.04	0.99	0.89	0.96
4-3	0.82	1.07	0.84	1.05
4-4	1.2	0.76	1.08	0.75
4-5	0.86	0.7	0.9	0.95
4-6	1.2	0.77	1.12	0.79
